# LigaSure Impact™ reduces complications after abdominoplasty in weight loss patients

**DOI:** 10.1007/s00423-021-02299-2

**Published:** 2021-08-31

**Authors:** Sonia Radunz, Haider Salem, Philipp Houben, Andreas Pascher, Martin Büsing, Markus Utech

**Affiliations:** 1 Department of General, Visceral and Transplant Surgery, University Hospital Münster, Albert-Schweitzer-Campus 1, 48149 Münster, Germany; 2 Department of General, Visceral and Bariatric Surgery, Plastic Surgery, Knappschaftskrankenhaus Recklinghausen, Recklinghausen, Germany; 3 Department of General and Visceral Surgery, Knappschaftskrankenhaus Bergmannsheil-Buer, Gelsenkirchen, Germany

**Keywords:** Abdominoplasty, Complications, Diathermia, Duration of surgery, LigaSure Impact™

## Abstract

**Purpose:**

Bariatric surgery is on the rise worldwide. With the desired weight loss after bariatric surgery, patients frequently develop massive skin flaps resulting in the need of abdominoplasty. In these patients, this surgical technique is frequently associated with perioperative complications. Strategies to minimize complications are sought after. The objective of our study was to compare two different dissection techniques and their impact on postoperative outcome.

**Methods:**

We included 66 patients in our study who underwent abdominoplasty after massive weight loss following bariatric surgery. In group 1, abdominoplasty was performed using the conventional technique of diathermia (*n* = 20). In group 2, abdominoplasty was performed using LigaSure Impact™ (*n* = 46). The duration of the surgical procedure and perioperative complications were recorded as primary endpoints. Secondary endpoints were length of hospital stay and assessment of additional risk factors.

**Results:**

Baseline characteristics were comparable between groups. The duration of surgery was significantly shorter in group 2. Postoperative complications were significantly less frequent in group 2 (*p* = 0.0035). Additional risk factors, e.g., smoking and diabetes mellitus, were not associated with increased rates of perioperative complications.

**Conclusions:**

The choice of technical device for dissection in abdominoplasty alone will not guarantee minimized complication rates. Yet, the utilization of LigaSure Impact™ in refined surgical techniques may facilitate reduced rates of complications, especially wound infections, and a shortened duration of surgery.

## Introduction

Abdominoplasty is an increasingly common plastic surgery performed in patients who had undergone bariatric surgery. Weight loss after bariatric surgery reduces the medical risks of obesity, but functional and psychosocial problems often remain due to the surplus skin with functional and esthetic impairments [1]. Patients benefit from abdominoplasty with improved quality of life, body satisfaction, and most importantly regained physical activity to sustain weight loss.

Especially in patients with massive weight loss after bariatric surgery, abdominoplasty has been associated with significant perioperative complications. When comparing patients with post-bariatric surgery weight loss to patients with weight loss due to dietary changes and exercise, a 60–87% increased risk of complications was demonstrated [2]. Seroma formation is the most common complication following abdominoplasty [3]. Hematoma, surgical site infection, wound dehiscence, and necrosis are frequently observed as well [4].

The surgical technique of abdominoplasty has continuously been refined to attain lower complication rates and improved outcomes. Hemostasis remains critical in preventing most perioperative complications. Previous studies comparing the initially applied devices, i.e., scalpel versus diathermia, did not universally demonstrate superior results for either device [5–7].

LigaSure Impact™ is an electrosurgical bipolar vessel sealing device fusing and dividing tissues with mechanic pressure and energy; it seals vessels up to 7 mm of diameter and delivers automatically the right amount of energy and time for tissue sealing as it detects tissue thickness [8]. Its application in general surgery, colorectal, urological, and gynecological procedures is associated with reduced operative time and fewer complications [9]. In abdominoplasty, reduced blood loss and fewer complications were observed when LigaSure Impact™ was applied; however, duration of surgery was significantly longer [10].

The objective of our study was to assess the duration of surgery and postoperative complications in abdominoplasty performed with conventional dissection techniques or LigaSure Impact™. We hypothesized that the application of LigaSure Impact™ can reduce the rate of perioperative complications as well as duration of surgery.

## Material and methods

Data on 66 patients who underwent abdominoplasty after bariatric surgery between January 2011 and December 2012 were retrospectively analyzed. The study was approved by the local ethics committee and was conducted in accordance with the Helsinki Declaration of 1975, as revised in 2008.

This study included patients who experienced weight loss due to bariatric surgery resulting in sagging abdominal skin folds. These skin folds lead to local inflammation and restriction of patient movement. All indications were peer-reviewed. Each surgery was performed with the prior approval of the health insurance company. Patients were instructed to stop or greatly reduce smoking 14 days prior to surgery. Whether the patients followed these instructions was not verified.

All patients underwent full abdominoplasty with umbilical transposition, conducted by three different general surgeons highly experienced in performing abdominoplasty, generally supported by two assistants [11]. A midline plication of the abdominal wall or a Fleur-de-Lis abdominoplasty was not performed. Generally, the skin incision was set in both groups along the preoperative markings by scalpel. The cutaneous incision line was drawn symmetrically in a crescent shape starting from laterally just below the costal arches both sides up to 5 cm suprapubic. In both groups prior to skin incision, the subcutaneous fat tissue was evenly infiltrated with 1 l of tumescence using a pump (PMID, 10,077,095). The dissection of the cutaneous flap up to xiphoid process was performed either with diathermia and ligation of transfascial veins by using 2–0 USP (United States Pharmacopeia) monofilament absorbable puncture-sutures or LigaSure Impact™; apart from that, there were no differences in surgical technique between the groups.

The scarpa fascia was preserved, and the subcutaneous tissue was dissected on the scarpa fascia to protect the lymphatic vessels. The umbilicus was excised for transposition. To establish the proximal resection line, the upper body was slightly elevated, and the skin flap was placed against the lower wound margin. Hernia was not observed in any patient. The weight of the removed excess skin was obtained. Two cross-perforated Redon drains (14 Charrière) attached to high-vacuum wound drainage systems were inserted. After temporary re-insertion of the umbilicus, the deep layers were closed with running sutures from medial to lateral on both sides using absorbable polyfilament sutures, i.e., Vicryl 1 CTX. The knots were placed extracorporeally and dissolved in due time. Subcutaneously and for skin closure absorbable monofilament sutures, i.e., monocryl 2–0, were placed. The previously clamped umbilicus was then sutured in place with 4–0 USP absorbable monofilament sutures using a single button technique. Finally, several layers of steri-strips and a sterile wound dressing were applied. Neither progressive tension sutures nor fibrin glue was used. Intraoperatively, a single intravenous dose of antibiotics was administered.

The starting point of incision and the endpoint of suturing were determined in a standardized manner as operation time. These periods were electronically documented. Patients were encouraged to mobilize on the first postoperative day. Drains were removed on the fourth postoperative day, and patients wore a support bandage for 14 days.

Postoperative seroma were generally treated by puncture at first. If patients presented with extremely large collections or recurrent seroma, surgical revision was performed. Wound infections were generally opened at the bedside. If further debridement was necessary, patients underwent surgical intervention with or without general anesthesia. Bedside treatment was graded as Clavien-Dindo grade I, and surgical intervention was graded as Clavien-Dindo grade IIIa/IIIb.

In group 1 (2011), abdominoplasty was performed using diathermia (*n* = 20). In group 2 (2012), abdominoplasty was performed using LigaSure Impact™ (*n* = 46). The indication for either device was *not* at the discretion of the individual surgeon. Baseline patients’ demographics (i.e., age, gender, weight, weight loss, risk factors) and perioperative data (i.e., duration of surgery, complications, length of hospital stay, readmission) were collected and analyzed.

Data collection and statistical analysis were performed using Microsoft Excel 2013 (Microsoft Corporation, Redmond, WA, USA) and GraphPad Prism version 6.07 for Windows (GraphPad Software, San Diego, CA, USA). All data were tested for normality using the D'Agostino and Pearson omnibus normality test. Categorical variables are presented as percentages and continuous variables as median (range), unless stated otherwise. Differences between categorical variables were tested using Fisher’s exact test or chi-square test as appropriate. Differences in continuous variables were tested using student’s *t* test or Mann–Whitney test as appropriate. A *p* value ≤ 0.05 (two-tailed) was considered to be significant.

Variables clinically relevant to the development of perioperative complications were included in a binary logistic regression model to estimate the impact of selected variables on the development of perioperative complications following abdominoplasty. All analyses have to be regarded as exploratory as we did not adjust the significance level globally in terms of the multiple testing problem. Final variables incorporated in the logistic regression model included patient age, gender, weight at the time of abdominoplasty, weight loss following bariatric surgery, smoking habit, diabetes mellitus, and surgical device. Logistic regression analysis was performed using IBM SPSS Statistics (version 27.0 for Windows, SPSS, Inc., Chicago, IL, USA).

## Results

Baseline characteristics were comparable between both groups (Table 1). Weight prior to bariatric surgery, weight loss after bariatric surgery, and weight at the time of abdominoplasty were comparable between groups. Time from bariatric surgery to abdominoplasty was comparable between groups as well. Risk factors typically associated with an increased risk for perioperative complications, i.e., smoking and diabetes mellitus, were equally present in both groups.

**Table 1:** Baseline patients’ characteristics

	Group 1 (*n* = 20)	Group 2 (*n* = 46)	*p*
Female gender (%)	60	69.6	0.5713
Age (yrs)	46.5 ± 7.4	46.7 ± 10.2	0.9299
Weight at bariatric surgery (kg)	155.0 [120.0–279.0]	151.5 [84.0–244.0]	0.6328
Weight at abdominoplasty (kg)	95.0 [71.0–175.0]	90.1 [63.0–165.0]	0.6804
Weight loss after bariatric surgery (kg)	60.5 ± 16.2	58.5 ± 20.6	0.7187
BMI at abdominoplasty (kg/m2)	30.5 [24.3–45.1]	32.1 [23.1–54.5]	0.9862
Smoking (%)	20.0	21.7	1.0000
Diabetes mellitus (%)	25.0	17.4	0.5117
Time to abdominoplasty (months)	15.0 [9.0–31.0]	18.0 [7.0–1281.0]	0.2765

**Fig. 1:**
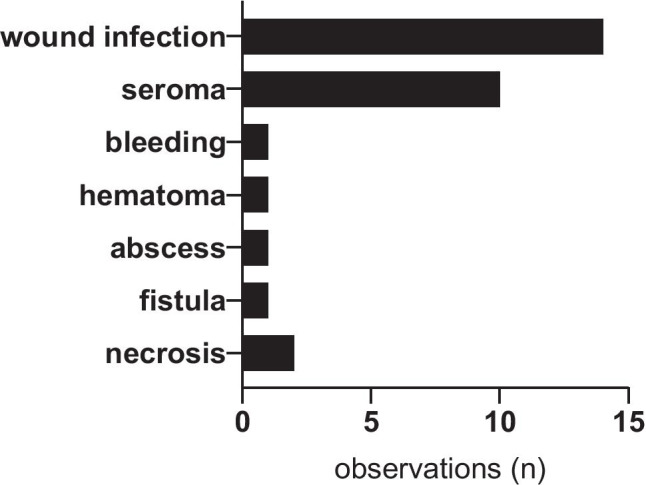
Overall perioperative complications

Perioperative complications were significantly more frequent in patients in group 1 compared to group 2 (55% (*n* = 11) vs. 19.6% (*n* = 9), *p* = 0.0077). Overall, 30 complications were documented in both groups (Fig. 1). Most frequent among these complications were wound infections (46.7%, *n* = 14) and seromas (33.3%, *n* = 10). Overall, readmission rate was 21.2% (*n* = 14). Patients with seroma were treated as outpatients in 60% (*n* = 6) and had to be readmitted for further treatment in 40% (*n* = 4), while patients with wound infections had to be readmitted in 71.4% (*n* = 10), and outpatient treatment was possible in 28.6% (*n* = 4). Differences between both groups regarding these types of complication are presented in Table 2. Clavien-Dindo grade III complications were significantly more frequent in group 1 (*p* = 0.0217).

**Table 2:** Most frequent perioperative complications

	Group 1 (*n* = 20)	Group 2 (*n* = 46)	*p*
Seroma (%)	15	15.2	1.0000
Requiring readmission (%)	10	4.35	0.5788
Wound infection (%)	45	10.9	**0.0034**
Requiring readmission (%)	40	4.4	**0.0007**
Clavien-Dindo grade I (%)	15	6.5	0.3570
Clavien-Dindo grade III (%)	40	14.2	**0.0217**

The entries in bold face do have significance, i.e. *p* < 0.05

Smoking habit did not significantly increase the rate of perioperative complications (smokers 42.9% versus non-smokers 23.1%, *p* = 0.1800). In group 1, complications occurred in 50% (*n* = 2) of smokers and in 43.8% (*n* = 7) of non-smoking patients (*p* = 1.0000). In group 2, complications occurred in 40% (*n* = 4) of smokers and in 13.9% (*n* = 5) of non-smoking patients (*p* = 0.0868). The pre-existing condition of diabetes mellitus did not significantly increase the rate of perioperative complications either (diabetics 15.4% vs. non-diabetics 34.0%, *p* = 0.3139). In group 1, complications occurred in 20% (*n* = 1) of diabetic patients and in 66.7% (*n* = 10) of non-diabetic patients (*p* = 0.0635). In group 2, complications occurred in 12.5% (*n* = 1) of diabetic patients and in 21.1% (*n* = 8) of non-diabetic patients (*p* = 1.0000).

The duration of abdominoplasty surgery was significantly longer in group 1 compared to group 2 (140.0 [88.0–191.0] *vs.* 108 [75–230] min, *p* = 0.0032). The length of hospital was shorter in group 2; however, this did not reach statistical significance (7.5 [4–52] *vs.* 6.0 [2–86] days, *p* = 0.0511).

Multivariate analysis detected body weight at the time of abdominoplasty and smoking habit as independently associated with increased odds of perioperative complications (Table 3). The choice of surgical device, i.e., LigaSure Impact™, was the only factor independently associated with decreased odds of perioperative complications following abdominoplasty.

**Table 3:** Results from multivariable logistic regression analysis of factors independently associated with perioperative complications

	Odds ratio (OR)	95% confidence interval (CI)	*p*
Age (per 10 yrs.)	1.859	0.0706, 4.893	0.209
Female gender	5.560	0.837, 36.930	0.076
Weight (per 10 kg)	2.077	1.335, 3.233	**0.001**
Weight loss (per 10 kg)	1.412	0.837, 2.383	0.196
Smoking habit	7.297	1.241, 42.918	**0.028**
Diabetes mellitus	0.100	0.007, 1.351	0.083
LigaSure Impact™	0.108	0.022, 0.523	**0.006**

The entries in bold face do have significance, i.e. *p* < 0.05

## Discussion

Abdominoplasty in itself as well as patients undergoing the procedure carry significant risk factors for perioperative complications. While the surgical technique has been constantly refined, complication rates are still considerable [12]. Therefore, it remains quintessential to define the most effective treatment mode with regard to individual safety and outcome as well as societal economic burden. In times of an increasing prevalence of obesity requiring bariatric surgery, the demand for post-bariatric abdominoplasty will be further rising.

The most common complications after abdominoplasty remain wound infections and seromas [13]. The overall complication rate in our study is comparable to previously published data reporting a total complication rate of 32.6% in abdominoplasty and a seroma rate of 15.4% [3]. In our study, we could significantly reduce the overall rate of perioperative complications when the LigaSure Impact™ device was applied. We detected mainly wound infections and seromas as well; especially wound infections were significantly less frequent when dissection was performed using the LigaSure Impact™ device. So far, only few studies analyzed the effectiveness of LigaSure Impact™ in plastic surgery demonstrating reduced rates of complications [14, 15]. In a single-center Finnish study comparing electrothermal devices for abdominoplasty, complications occurred significantly less frequent when using LigaSure Impact™ but with a higher rate of 34.5% than in our study [10]. To prevent abdominoplasty-related complications, preoperative hyperbaric oxygen therapy has been applied with a significant reduction in postoperative complications [16].

Previous studies highlighted various risk factors for complications in abdominoplasty, i.e., age, obesity, smoking, weight loss, and blood loss [10, 17]. In our study, smoking habit and body weight at the time of abdominoplasty, but not weight loss since bariatric surgery, were independently associated with increased odds of perioperative complications following abdominoplasty. As smoking habits are only assessed by patient survey, they may be underestimated among any study population. The presence of diabetes mellitus was rather low with 19.7% of patients in our study. Therefore, the ability to detect small differences between groups and to adjust for all potential confounders is limited. In our study population, age was not identified as a potential risk factor. This is in accordance with data by Couto et al.; they did not find any statistically significant differences in major, minor, local, or systemic complications when age groups of less or greater than 60 years were compared [18]. As most surgical procedures, abdominoplasty may safely be performed regardless of age in the setting of a proper patient selection.

The duration of surgery was significantly shortened by approximately 30 min when using the LigaSure Impact™ device in our study. This has been proven for many surgical procedures. To avoid severe bleeding from the transfascial veins when using diathermia for dissection, these veins were selectively visualized, and transfixing ligatures were applied. This elaborate preparation requires more time than using the LigaSure™ device. In total thyroidectomy, the use of LigaSure™ significantly reduced operation time [19]. In an early meta-analysis, operative time was reduced in 24 of 26 studies of different surgical procedures when an electrothermal bipolar vessel sealing device (EBVS) was used as compared with conventional surgical hemostasis; the normalized mean operative time reduction for EBVS equaled 28% [9]. By contrast, operative time was longer with LigaSure Impact™ in a recent study among patients undergoing abdominoplasty; the authors accounted the time of vessel sealing by the device for the extended duration of surgery [10]. In plastic surgery in general, the duration of surgery has been proven as an independent predictor of complications with a significantly increased risk in surgeries of more than 3 h [20]. Shortened operative time results in shortened anesthesia time, which may be beneficial for the individual patient. Furthermore, shortened operative time may grant additional capacity for treating other patients.

The length of hospital was shorter when using the LigaSure Impact™ device in our study; however, this difference did just not reach statistical significance. A recent study among patients undergoing abdominoplasty did not detect significant differences in length of hospital either [10]. Yet, it is noteworthy that length of hospital stay in Finland (3.6–4.6 days) was shorter than in our study (6.0–7.5 days).

A disposable device always constitutes additional costs; nevertheless, it may be cost-saving due to shortened surgery duration and decreased complication rates requiring outpatient treatments or hospital readmissions. In our study, the choice of surgical device, i.e., LigaSure Impact™, was the only factor independently associated with decreased odds of perioperative complications following abdominoplasty.

Despite the significant findings, there are some limitations to our study. This is a single-center study carrying the risk of bias for treatment. In a retrospective setting, any causal relationship between type of device and outcome of surgery cannot be confirmed. Many confounders may occur in the analysis of perioperative outcome of abdominoplasty patients. Only a prospective randomized controlled trial could prove the presented benefits of the application of a surgical device.

In conclusion, the choice of technical device for dissection in abdominoplasty alone will not guarantee minimized complication rates. Yet, the utilization of LigaSure Impact™ in refined surgical techniques may facilitate reduced rates of complications, especially wound infections, and a shortened duration of surgery.
